# Population genetic structure of wolves in the northwestern Dinaric‐Balkan region

**DOI:** 10.1002/ece3.8444

**Published:** 2021-12-12

**Authors:** Dragana Šnjegota, Astrid Vik Stronen, Barbara Boljte, Duško Ćirović, Mihajla Djan, Djuro Huber, Maja Jelenčič, Marjeta Konec, Josip Kusak, Tomaž Skrbinšek

**Affiliations:** ^1^ Department of Biology and Ecology Faculty of Natural Sciences and Mathematics University of Banja Luka Banja Luka Bosnia and Herzegovina; ^2^ Department of Biology Biotechnical Faculty University of Ljubljana Ljubljana Slovenia; ^3^ Faculty of Biology University of Belgrade Belgrade Serbia; ^4^ Department of Biology and Ecology Faculty of Sciences University of Novi Sad Novi Sad Serbia; ^5^ Department of Biology Faculty of Veterinary Medicine University of Zagreb Zagreb Croatia

**Keywords:** Balkan Peninsula, *Canis lupus*, Dinaric Mountains, effective population size, microsatellites, population structure

## Abstract

The Balkan Peninsula and the Dinaric Mountains possess extraordinary biodiversity and support one of the largest and most diverse wolf (*Canis lupus*) populations in Europe. Results obtained with diverse genetic markers show west‐east substructure, also seen in various other species, despite the absence of obvious barriers to movement. However, the spatial extent of the genetic clusters remains unresolved, and our aim was to combine fine‐scale sampling with population and spatial genetic analyses to improve resolution of wolf genetic clusters. We analyzed 16 autosomal microsatellites from 255 wolves sampled in Slovenia, Croatia, Bosnia and Herzegovina (BIH), and Serbia and documented three genetic clusters. These comprised (1) Slovenia and the regions of Gorski kotar and Lika in Croatia, (2) the region of Dalmatia in southern Croatia and BIH, and (3) Serbia. When we mapped the clusters geographically, we observed west‐east genetic structure across the study area, together with some specific structure in BIH–Dalmatia. We observed that cluster 1 had a smaller effective population size, consistent with earlier reports of population recovery since the 1980s. Our results provide foundation for future genomic studies that would further resolve the observed west‐east population structure and its evolutionary history in wolves and other taxa in the region and identify focal areas for habitat conservation. They also have immediate importance for conservation planning for the wolves in one of the most important parts of the species’ European range.

## Graphical abstract

In this manuscript, we analyzed microsatellites from 255 wolves sampled in Slovenia, Croatia, Bosnia and Herzegovina (BIH), and Serbia and documented three genetic clusters as follows: (1) Slovenia and the regions of Gorski kotar and Lika in Croatia, (2) the region of Dalmatia in southern Croatia and BIH, and (3) Serbia. When we mapped the clusters geographically, we observed west‐east genetic structure across the study area, together with some specific structure in BIH—Dalmatia. Our results provide foundation for future genomic studies that would further resolve the observed west‐east population structure and its evolutionary history in wolves and other taxa in the region and identify focal areas for habitat conservation. They also have immediate importance for conservation planning for the wolves in one of the most important parts of the species’ European range.

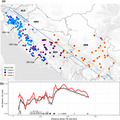

## 1 INTRODUCTION

Population genetic studies of European wolves (*Canis lupus*) have been conducted using various molecular markers (e.g., Djan et al., 2014; Montana et al., 2017; Pilot et al., 2006, 2010; Stronen et al., 2013). These studies have given us comprehensive insights into the historical and present‐day population structure of a species that once occurred across Europe. Nowadays, European wolves are divided into several populations (Kaczensky et al., 2013), with habitat loss, human–wildlife conflicts, hybridization with domestic dogs (*C*. *l*. *familiaris*), and other processes affecting the observed structure (Loxterman, 2011; Sinclair et al., 2001; Walker et al., 2002; Woodroffe & Frank, 2005). However, various changes, including the implementation of numerous management conservation programs in recent decades (Chapron et al., 2014), have allowed wolves to recolonize substantial parts of their former ranges and facilitated reconnection of previously separated populations (e.g., Louvrier et al., 2020; Nowak et al., 2016; Schley et al., 2021).

One of the largest wolf populations in Europe occupies the Dinaric‐Balkan region in the southeastern part of the continent (Chapron et al., 2014; Hindrikson et al., 2017). The Dinaric‐Balkan population thus inhabits a region that provided a major glacial refugia for many taxonomic groups (Hewitt, 2000) and retains a substantial amount of the historical genetic variation of the species at the continental level (e.g., Gomerčić et al., 2010; Randi et al., 2000). The population plays a key role in connecting eastern and western European wolves (Djan et al., 2014) and represents an important source of genetic diversity for the long‐isolated Italian population (Fabbri et al., 2014; Ražen et al., 2016) and possibly also for areas further north, including Germany (Bayerishes Landesamt für Umwelt, 2021). Previous population genetic studies with different genetic markers, including mitochondrial DNA (mtDNA) (Djan et al., 2014; Šnjegota, 2019), microsatellites (Fabbri et al., 2014; Montana et al., 2017; Šnjegota, 2019), and single nucleotide polymorphisms (SNPs) (Stronen et al., 2013), showed a consistent west‐east gradient in the Dinaric‐Balkan population. However, these studies were mostly conducted locally or included broader areas with discontinuous sampling. For example, genome‐wide SNP analyses found differentiation between wolves sampled in Bulgaria and Greece versus profiles from Croatia (Stronen et al., 2013), but because of discontinuous sampling, it was not possible to determine the spatial extent of these population clusters. Additional fine‐scale sampling is therefore required to determine the substructure of the broader Dinaric‐Balkan population. Moreover, Djan et al. (2014) observed western and eastern subpopulations based on mtDNA haplotypes, with Bosnia and Herzegovina (henceforth BIH) and Croatia divergent from Serbia and North Macedonia, suggesting that more detailed sampling and analysis of molecular markers of higher resolution across this region can help resolve population structure and provide a baseline for genetic monitoring and vital transboundary conservation efforts.

The recolonization of wolves across Europe creates potential conflicts with humans and thus requires the establishment or modification of conservation measures and national legislation for this species (Salvatori et al., 2020). These issues are particularly important for the Dinaric‐Balkan population, which spans several national borders and differences in national legislation (Hindrikson et al., 2017), and where so far, only Croatia and Slovenia have enacted conservation legislation for wolves. The aim of our study was to combine continuous fine‐scale sampling of wolves with comprehensive population and spatial genetic analyses and use this to better understand regional genetic structure for this species, providing solid background for species conservation.

## 2 MATERIALS AND METHODS

### 2.1 Sample collection and study area

**FIGURE 1:**
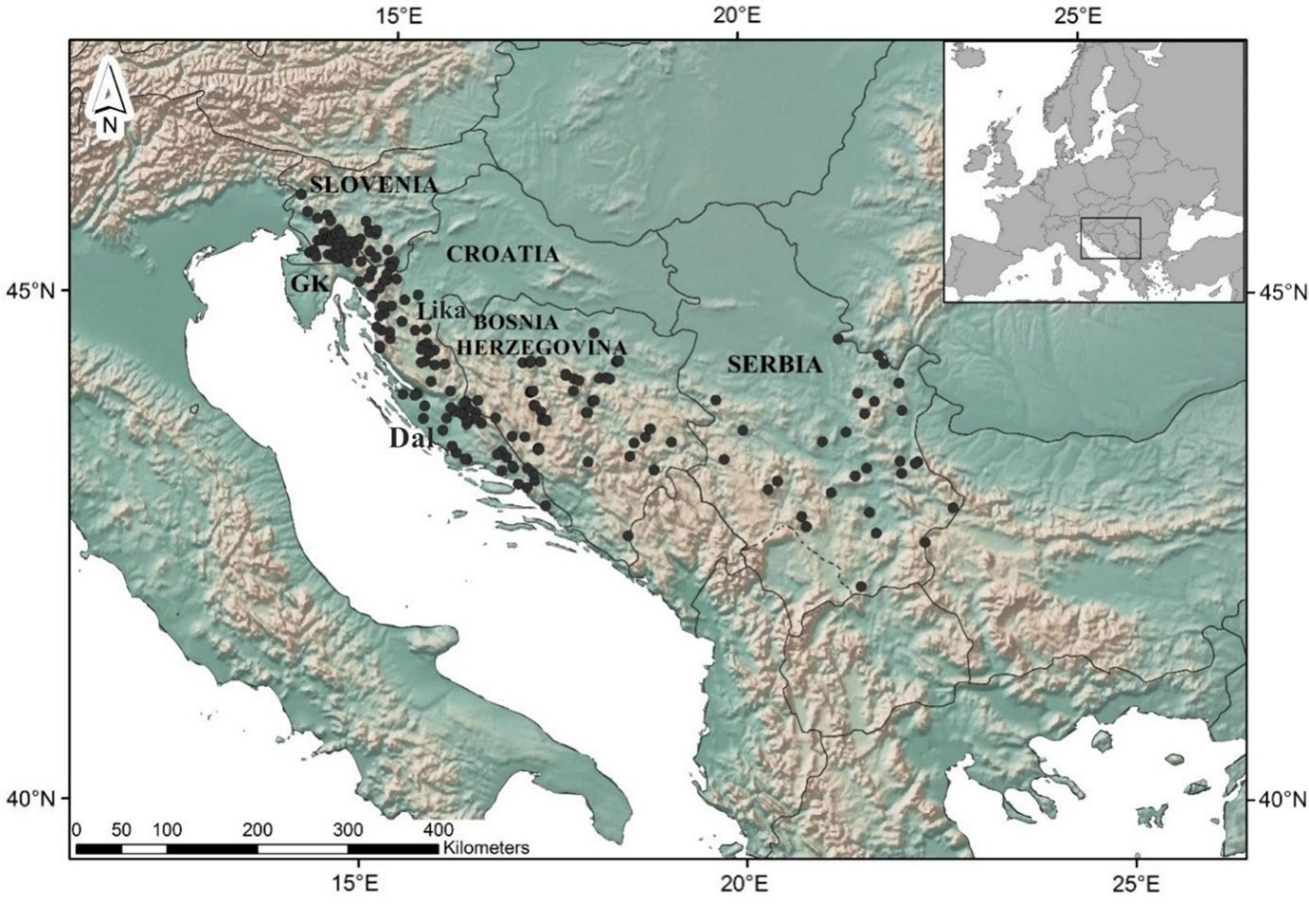
Map showing sampling localities of wolves from Slovenia, Croatia, Bosnia and Herzegovina, and Serbia, including the three regions of Croatia (Dal, Dalmatia; GK, Gorski kotar; Lika)

Sampling was conducted in Slovenia (*n* = 65), Croatia (*n* = 94), BIH (*n* = 59), and Serbia (*n* = 37) (Figure 1; Appendix S1: Note S1), broadly comprising most of the wolf range in these countries (Chapron et al., 2014; Kaczensky et al., 2013). Organization of Croatian wolves into three subgroups (Gorski kotar, Lika, and Dalmatia) was done based on previous results showing regional substructure (Fabbri et al., 2014). The initial screening (Appendix S1: Figure S1) resulted in 255 individual profiles for further analyses; 249 samples were collected during 2010–2018, whereas six were collected before 2010. The majority of BIH samples were examined for population genetic structure by Šnjegota et al. (2018) (Appendix S1: Figure S2), and these samples were regenotyped for this study to ensure compatible genotypes. Samples were tissues of individuals found dead from various causes, and no animal was killed for the purpose of this study. After sampling, tissues were preserved in 95% ethanol and stored at ‒20°C.

### 2.2 DNA extraction, microsatellite amplification, and genotyping

DNA extraction for the wolf muscle tissue samples from (i) BIH and Serbia was performed using a phenol–chloroform DNA isolation method Sambrook & Russell, 2001) and (ii) Slovenia and Croatia using the GeneElute Mammalian Genomic DNA Miniprep Kit (Sigma‐Aldrich) following the manufacturer's protocol, as well as hair samples of dogs (Appendix S1: Note S1), with overnight extended proteinase K digestion step at 56°C (Karamanlidis et al., 2010).

Sixteen autosomal microsatellite loci (AHT137, AHTh171, AHTh260, AHTk211, AHTk253, CXX279, FH2054, FH2848, INRA21, INU030, INU005, REN162C04, REN169D01, REN169O18, REN247M23, REN54P11) and the sex marker Amelogenin were amplified using The Canine Genotypes™ Panel 1.1 kit (Finnzymes, Thermo Fisher Scientific) following the manufacturer's recommendations and Pedersen et al. (2012). Genotyping was performed on an ABI 3130xl Genetic Analyzer (Applied Biosystems) for all samples at the Biology Department, Biotechnical Faculty, University of Ljubljana, Slovenia, and the size of each allele was determined by GENEMAPPER V.4 (Applied Biosystems). All genotypes were independently called twice with the aim of: (i) confirming allele calls, (ii) checking the occurrence of allelic dropout, and (iii) identifying false alleles.

### 2.3 Data analysis

#### 2.3.1 Detection of wolf population structure after the initial screening

Genetic clustering analysis was performed using STRUCTURE v2.3.4 (Falush et al., 2003, 2007; Hubisz et al., 2009; Pritchard et al., 2000). The 255 wolf samples were divided into six a priori determined groups, according to the country (region) of sampling: Slovenia, Gorski kotar, Lika, Dalmatia, BIH, and Serbia. Simulations were run with an MCMC of 10^6^ iterations after a burn‐in of 10^5^ by applying the population admixture model with correlated allele frequencies. We set the number of population clusters (*K*) from 1 to 10, and we did 10 independent runs for each *K*, without the LOCPRIOR setting. We assessed the most likely *K*‐value based on log likelihoods [ln Pr(*X*|*K*)] and the Δ*K* method of Evanno et al. (2005) in Structure Harvester (Earl & vonHoldt, 2012). Based on the selected *K*‐value, we determined individual membership (*q*) and the average membership (*Q*) in each cluster with a q‐value threshold of 0.10. The interpretation of results from multiple runs of each *K*‐value was done by CLUMPAK (Kopelman et al., 2015). Spatial population structure was examined with QGIS v.3.4.11 (QGIS Development Team, 2018) to help determine the optimal *K*‐value.

We further explored genetic structure using discriminant analysis of principal components (DAPC) (Jombart et al., 2010). The method applies discriminant analysis to principal components (PCA), maximizing variation between groups and minimizing variation within groups, effectively achieving the best structuring of individuals in predefined groups. This approach does not rely on explicit population genetic models like Bayesian clustering methods and is thus more robust to assumptions of equilibrium conditions and more suitable for identifying spatial patterns such as genetic clines. We used the R package ADEGENET v2.1.1 for the analysis (Jombart, 2008; Jombart & Ahmed, 2011) in the R 3.5.3. environment (R Core Team, 2019). We ran DAPC twice, defining groups de novo and using geographic locations as a priori groups (Miller et al., 2020). In de novo analysis, groups were defined using *K*‐means clustering, and the *K* value with the lowest Bayesian information criterion (BIC) was selected as optimal. We used cross‐validation to determine the optimal number of principal components retained.

#### 2.3.2 Spatial structure

We also explored population structure in a spatially explicit manner via spatial principal component analysis (sPCA) in ADEGENET, summarizing spatial patterns of genetic structure by defining eigenvalues that optimize the product of the genetic variance and Moran's *I* (Moran, 1948, 1950). These patterns can be positive (i.e., global structure) and negative (i.e., local structure). Global patterns are used to identify clines in allele frequencies and genetically distinguishable groups, while local patterns detect differences between adjacent individuals (Jombart, 2008). We experimented with different methods to define the connection network (CN) and used the CN that seemed to best describe the spatial relationships between animals in visual examination. We ran the Monte Carlo test for the presence of local and global structure and examined plot of eigenvalues and sPCA scree plot to estimate the number of interpretable components.

We additionally performed a spatial autocorrelation analysis in GenAlEx v6.5 (Peakall & Smouse, 2012) to examine the possible existence of isolation‐by‐distance in our dataset. We used distance classes of 30 km to obtain fine‐scale results for our study area.

#### 2.3.3 Genetic variability

The mean number of alleles per locus (*N*_a_), the number of private alleles per cluster (*N*_p_), observed (*H*_o_) and expected (*H*_e_) heterozygosities, deviations from Hardy–Weinberg equilibrium (HWE) and linkage‐disequilibrium (LD), were calculated for the determined (consensus) clusters in GENALEX v6.5 (Peakall & Smouse, 2012), as well as the pairwise *F*_ST_ values and AMOVA. We used the STRUCTURE results for the selected *K*‐value to define consensus population clusters, based on the individual membership (*q*) and the average membership (*Q*) in each cluster with a *q*‐value threshold of 0.10. A sequential Bonferroni correction for multiple tests was applied (Rice, 1989).

To understand the distribution of genetic diversity and potential drivers of genetic differentiation that may be shaping it, we used Hardy–Weinberg Dynamic Subsampling analysis (HWDS) (Karamanlidis et al., 2018). We used the “Dinaric” NW–SE direction (Figure 3a) as the traveling window path and defined each “window” as 30 geographically consecutive genotypes. For each window, we calculated genetic diversity parameters and estimated effective population size (*N*_e_). We graphed these parameters in the context of distance along the traveling window axis to understand and interpret their interplay.

#### 2.3.4 Gene flow

We used the Bayesian method developed by Wilson and Rannala (2003) and implemented in BAYESASS v3.0.3 to infer gene flow between estimated population clusters. We adjusted the mixing parameters (i.e., the size of the proposed parameter change in each MCMC iteration) for allele frequencies, migration rates, and inbreeding coefficients to get the acceptance rates of proposed moves between 0.2 and 0.4 (following the program authors’ recommendation). We plotted trace files in R to visually check MCMC for adequate mixing and convergence. We ran 10 MCMC chains with different random seeds, 1.0E07 steps in each chain and 1.0E06 steps of burn‐in. We calculated the Bayesian deviance for each chain to estimate model fit (Faubet et al., 2007) using the R script provided by Meirmans (2014) and used the MCMC chain with the lowest deviance for the final estimates of the migration rates (Meirmans, 2014).

#### 2.3.5 Effective population size

To explore effective population size in the estimated population clusters and its distribution along the traveling window axis, we used the unbiased linkage disequilibrium estimator (Waples, 2006) in program NeEstimator (Do et al., 2014), excluding rare alleles below an allele frequency of 0.02 (following recommendations of Waples & Do, 2010). The linkage disequilibrium method tests for nonrandom associations formed among alleles at different loci that occur when *N*_E_ is low (Luikart et al., 2010), and the method is reasonably precise and unbiased in small populations already at sample sizes of 25 individuals (Waples, 2006; Waples & Do, 2010).

## 3 RESULTS

### 3.1 Detection of wolf population structure after the initial screening

**FIGURE 2:**
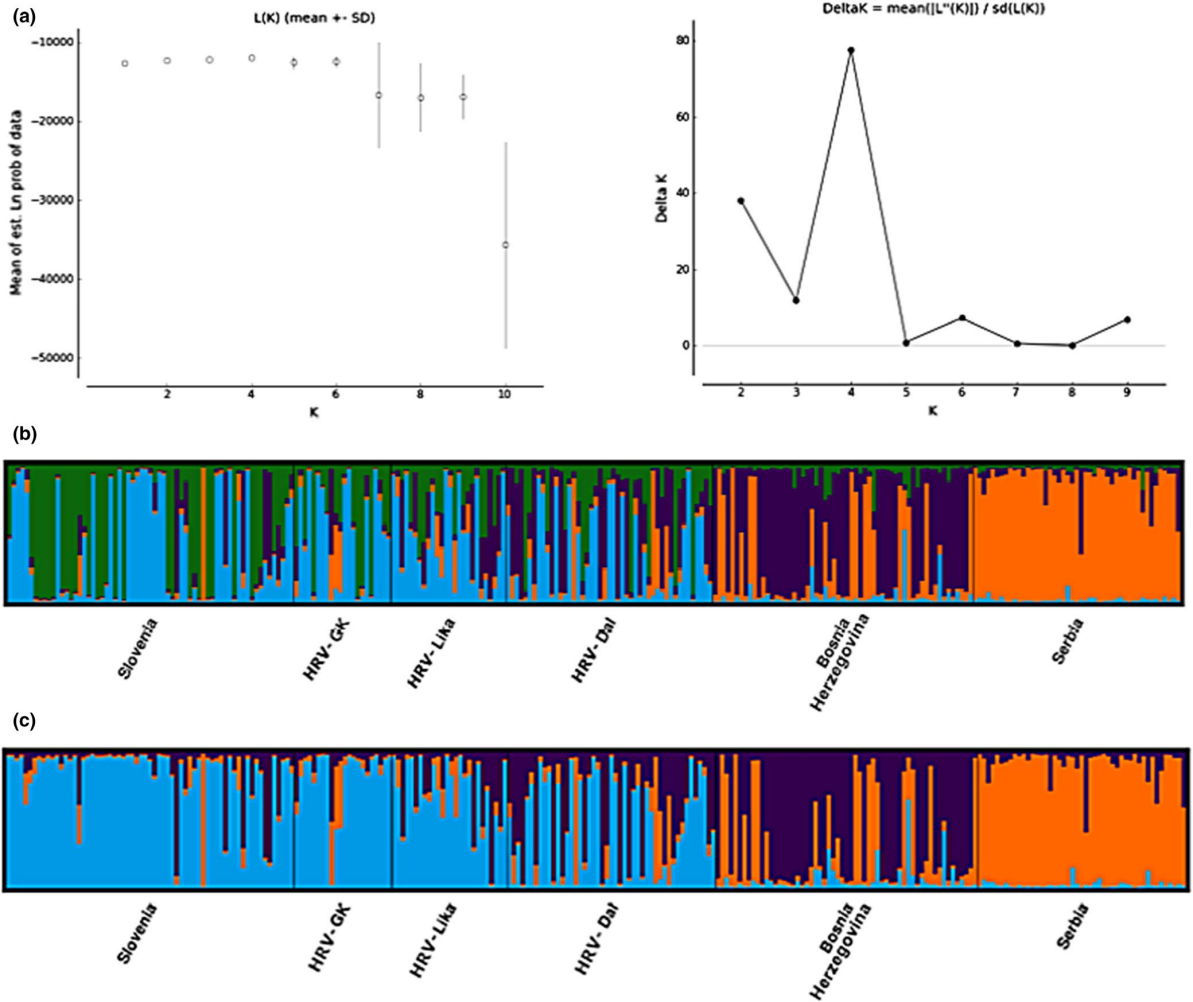
(a) The most likely number of clusters (*K* = 4) detected for wolves from Slovenia, Croatia, Bosnia and Herzegovina, and Serbia in STRUCTURE, based on log‐likelihoods [ln Pr(*X*|*K*)] and the Δ*K* method. (b) Bar plot from the STRUCTURE analyses showing four genetic clusters (*K* = 4). Each color corresponds to one cluster; each line represents one individual, showing probability of assignment (range 0–1) per cluster. (c) Bar plot from the STRUCTURE analyses showing three genetic clusters (*K* = 3)

**FIGURE 3:**
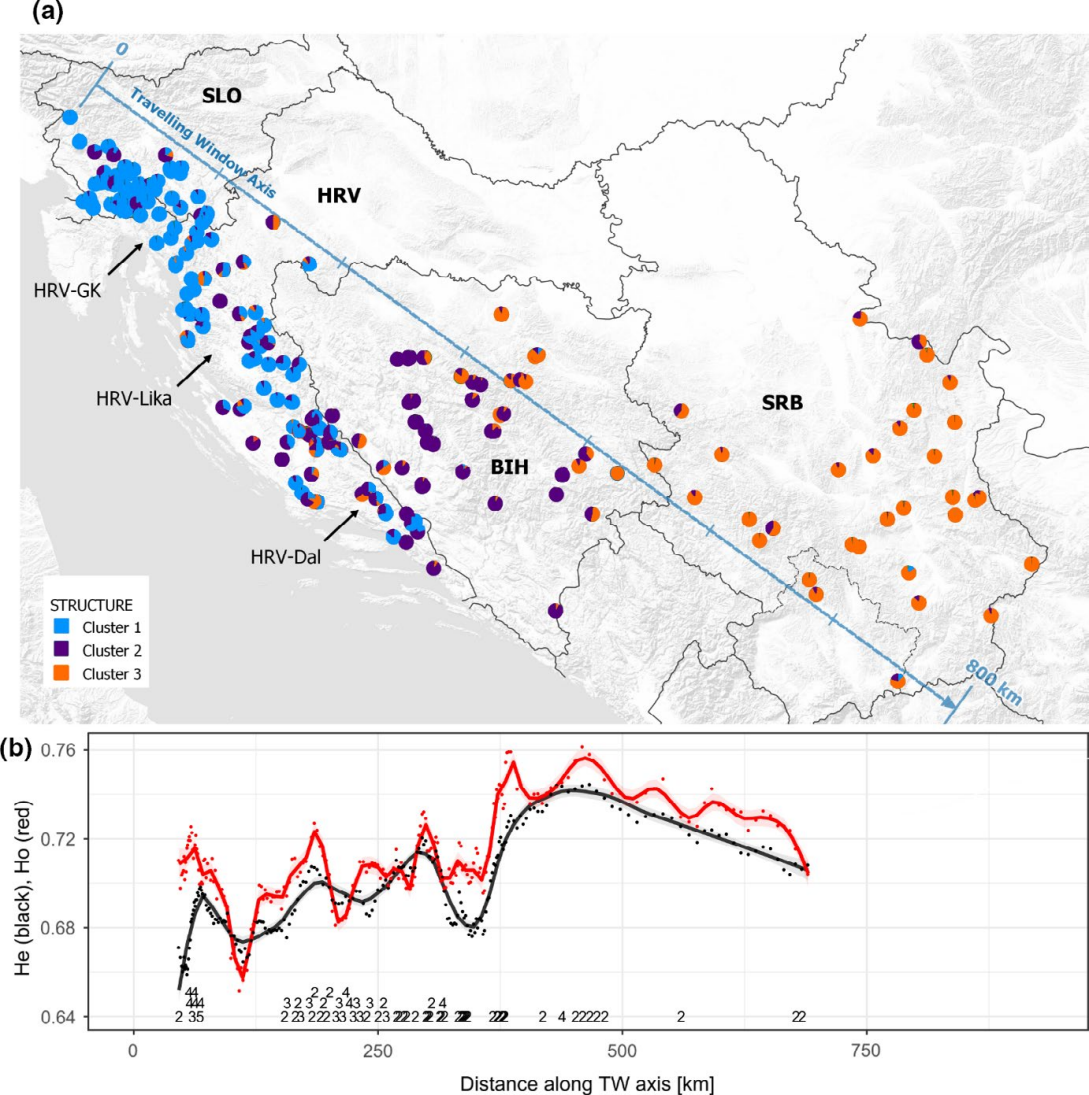
(a) The geographical distribution and observed west‐east gradient of wolf genetic clusters (*K* = 3) based on individual q‐values in STRUCTURE. Pie chart colors designate the individual membership assignment to each cluster. Abbreviations used are BIH, Bosnia and Herzegovina; HRV‐Dal, Croatia‐Dalmatia; HRV‐GK, Croatia‐Gorski kotar; HRV‐Lika, Croatia‐Lika; SLO, Slovenia; SRB, Serbia. (b) HWDS analysis. Distance coincides with the distance on the traveling window (TW) axis in (a). The values at the bottom indicate the number of loci deviating from Hardy–Weinberg expectations at *p *< .05. Some of the values were removed where the points are very dense (particularly between 0 and 100 km) to improve legibility

The STRUCTURE values of log likelihoods [ln Pr(*X*|*K*)] and Δ*K* suggested *K* = 4 as the most probable number of clusters (Figure 2a). However, after comparing the spatial distribution of the *K* = 4 (Figure 2b; Appendix S1: Figure S3) and *K* = 3 (Figures 2c and 3a) results, the distribution of individuals of this highly mobile species was better interpreted as *K* = 3 spatial population clusters, with some expected intergradation between neighboring units. Although *K* = 4 may explain more of the variation (Appendix S1: Figure S4), *K* = 3 provided a clearer spatial genetic structure and a more practical baseline for population management and conservation planning. We therefore present both outcomes, but adopted *K* = 3 as the most parsimonious result for the subsequent analyses.

According to the *q*‐values, 90 individuals (35%) formed cluster 1, while 50 individuals (19.5%) were assigned to cluster 2 and 45 (17.6%) to cluster 3. Seventy individuals (27.4%) were classified as admixed with contributions from different wolf population clusters. Most of the admixed wolves were sampled in BIH and in Dalmatia (Croatia). After plotting individuals according to their *q*‐values for *K* = 3 clusters on the map, a clear northwest–southeast gradient was visible (Figure 3a). The results showed three subpopulations generally corresponding to Croatia and Slovenia (cluster 1), BIH and Dalmatia (cluster 2), and Serbia (cluster 3), with some admixture among clusters.

A similar west‐east gradient was also visible from the PCA and DAPC results (Appendix S1: Figure S5). In PCA, the first principal component axis indicated differentiation of wolves from Slovenia, Gorski kotar, and Lika from those in Serbia. Wolves from BIH and Dalmatia represented the main area of admixture. DAPC provided a clearer picture than PCA, and the most stable and interpretable result was obtained with a priori groupings according to sampling location (Appendix S1: Figure S5B,C), with the de novo grouping analysis indicating a similar pattern but less clear (not shown). We ended up retaining 60 PCs, as suggested by the cross‐validation, but the testing of different numbers of retained PCs (from 20 to 100) provided a similar interpretation. DAPC showed a clear differentiation of animals sampled in Serbia and a gradient from Slovenia to BIH for other animals (Appendix S1: Figure S5B). When the sampling location groups were merged according to the clustering suggested by STRUCTURE, the result showed a well‐separated cluster in Serbia and two closer clusters in Slovenia–Croatia (Gorski kotar and Lika) and BIH–Croatia (Dalmatia) (Appendix S1: Figure S5C).

### 3.2 Spatial structure

**FIGURE 4:**
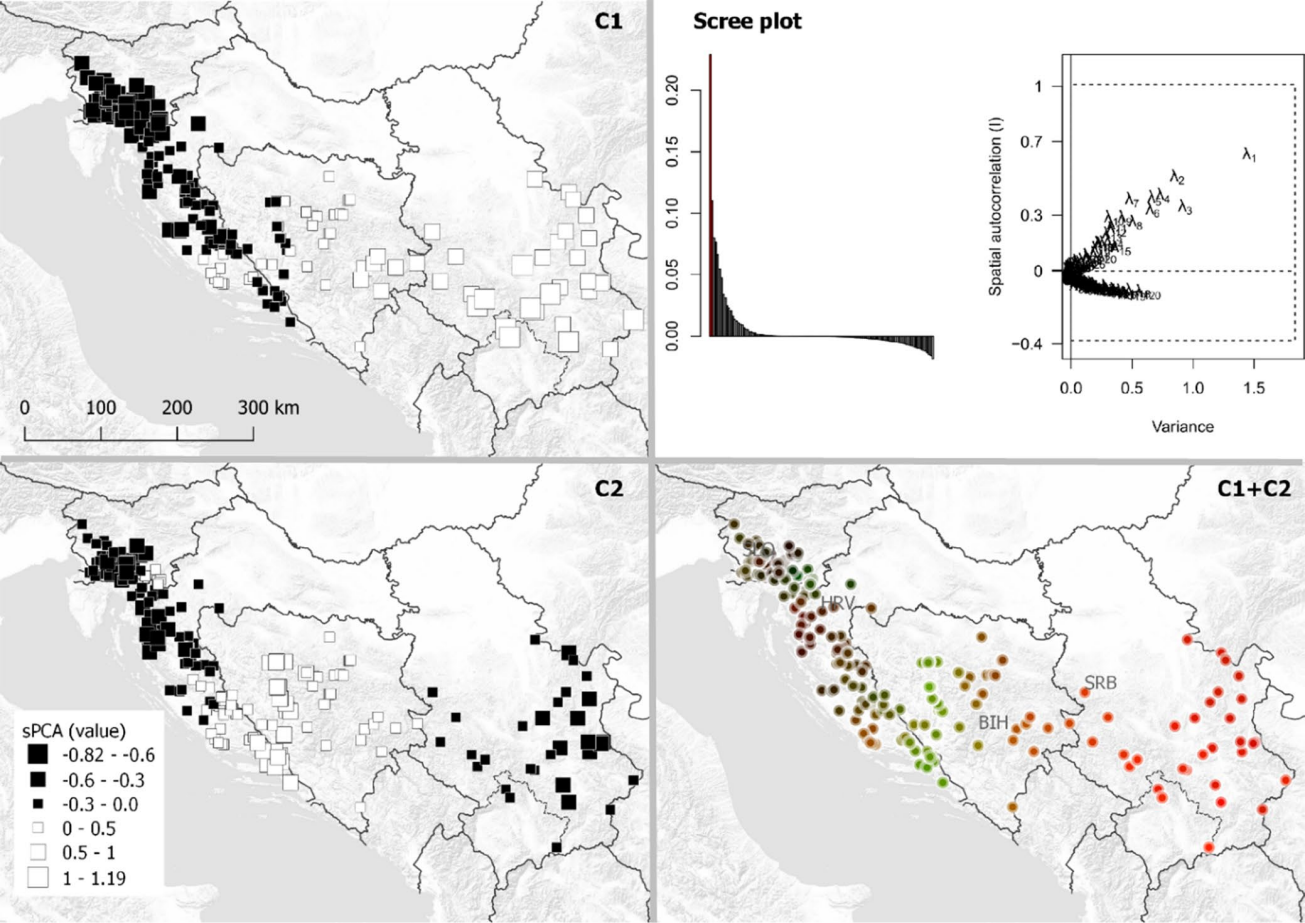
Spatial principal component analysis (sPCA) showing genetic population structure in the study area. C1, C2: Global sPCA components 1 and 2, the size and colors of squares indicate differences in value (between individuals) for the respective component; C1 + C2 – colorplot of sPCA components 1 and 2, each mapped to a RGB color (component 1 = red, component 2 = blue). Scree plot: left—barplot of the eigenvalues; right—spatial and variance components of the eigenvalues

For the sPCA, the tests indicated statistically significant global structure (*p* < .001) and no local structure (*p* = .395). Plot of eigenvalues and sPCA scree plot indicated two or possibly three interpretable components (Figure 4 Scree plot). When mapped geographically, the first component indicated a west–east cline, suggesting a clinical genetic structure in the study area (Figure 4C1). The second component (Figure 4C2), on the other hand, suggested some specific structure in BIH–Dalmatia. The third component did not indicate any clearly interpretable structure.

The results of a spatial autocorrelation analysis in GenAlEx (Appendix S1: Figure S6) also suggest the presence of genetic structure in the central part of our study area. These results were obtained with distance classes of 30 km, but we also performed the analyses with 50 and 100 km where we obtained similar results (not shown).

### 3.3 Genetic variability and gene flow

Analyses of wolf genetic variability showed that all loci were polymorphic. The highest mean values for the number of alleles (*N*_a_ = 8), observed (*H*_o_ = 0.74), and expected (*H*_e_ = 0.75) heterozygosities were detected for cluster 2, generally corresponding to wolves from BIH and Dalmatia, while the smallest mean number of alleles (*N*_a_ = 6) and values of heterozygosities (*H*_o_ = 0.69, *H*_e_ = 0.70) were detected in cluster 1, generally corresponding to wolves from Slovenia, Lika, and Gorski kotar. Loci deviating from HWE were found in cluster 1 and cluster 2 (Table 1). No locus showed significant LD after correction for multiple testing. The largest number of private alleles was detected in cluster 3, generally corresponding to wolves from Serbia (18 private alleles over eight loci); ten private alleles over seven loci were detected in cluster 2, and one private allele in cluster 1 (Appendix S1: Figure S7). The highest molecular variance (91%) among individuals (AMOVA, *p *< .001) and pairwise *F*_ST_ values were statistically significant (Appendix S1: Table S1).

**TABLE 1:** Basic parameters of genetic variability for wolves assigned to the consensus wolf genetic clusters (*K* = 3)

	Cluster 1 (SLO, HRV–GK, HRV–Lika; *N* = 90)				Cluster 2 (BIH, HRV–Dal; *N* = 50)				Cluster 3 (SRB; *N* = 45)			
	*N*_a_	*H*_o_	*H*_e_	HWE	*N*_a_	*H*_o_	*H*_e_	HWE	*N*_a_	*H*_o_	*H*_e_	HWE
AHT137	7	0.82	0.81	ns	10	0.81	0.80	ns	12	0.90	0.87	ns
AHTh171	5	0.70	0.70	ns	7	0.87	0.84	ns	8	0.82	0.80	ns
AHTh260	8	0.83	0.84	*	14	0.83	0.86	ns	8	0.85	0.86	ns
AHTk211	5	0.54	0.58	ns	5	0.74	0.68	ns	4	0.31	0.35	ns
AHTk253	5	0.73	0.76	ns	8	0.86	0.81	ns	7	0.77	0.73	ns
CXX279	7	0.76	0.76	ns	9	0.86	0.81	ns	7	0.70	0.71	ns
FH2054	7	0.65	0.57	ns	9	0.72	0.78	*	6	0.74	0.73	ns
FH2848	5	0.81	0.76	ns	5	0.68	0.72	ns	4	0.77	0.68	ns
INRA21	5	0.58	0.65	*	6	0.43	0.62	ns	5	0.68	0.67	ns
INU030	6	0.64	0.69	ns	6	0.69	0.67	ns	6	0.69	0.71	ns
INU005	5	0.61	0.73	*	6	0.59	0.62	ns	7	0.69	0.73	ns
REN162C04	5	0.59	0.53	ns	9	0.71	0.78	ns	5	0.60	0.56	ns
REN169D01	6	0.71	0.69	ns	8	0.68	0.74	*	8	0.81	0.81	ns
REN169O18	5	0.50	0.58	ns	7	0.81	0.79	ns	7	0.71	0.72	ns
REN247M23	7	0.90	0.84	ns	7	0.81	0.78	ns	8	0.79	0.80	ns
REN54P11	5	0.64	0.64	ns	7	0.76	0.71	ns	6	0.76	0.64	ns
Mean	6	0.69	0.70		8	0.74	0.75		7	0.73	0.71	
SD	1.05	0.12	0.10		2.24	0.12	0.07		1.95	0.13	0.13	

Note

*N*_a_, number of alleles per locus; *H*_o_, observed heterozygosity; *H*_e_, expected heterozygosity; HWE, Hardy–Weinberg equilibrium (ns, no statistically significant deviation from HWE; *p < .05—statistically significant deviation after sequential Bonferroni correction).

Abbreviations: BIH, Bosnia & Herzegovina; Dal, Dalmatia; GK, Gorski kotar; Lika, Lika; SLO, Slovenia; SRB, Serbia.

The HWDS analysis indicated some local departures from HWE in certain areas (Figure 3b) and mostly a west‐to‐east increase in heterozygosity. In most cases of detectable departure from HWE, the observed heterozygosity (*H*_o_) was higher than expected heterozygosity (*H*_e_). This indicates that the “isolate breaking” effect (Wahlund, 1928) of having recent‐generation offspring of immigrants causing heterozygote excess (*H*_o_ > *H*_e_) in the recipient population (cluster) overrides the impact of the Wahlund effect (*H*_e_ > *H*_o_). The Wahlund (1928) effect appears when sampling animals from different clusters and/or sampling direct immigrants in the recipient populations (clusters).

BAYESASS analysis showed higher geneflow between neighboring populations, compared to those further apart, with considerably higher migration rate from BIH–HRV (Dalmatia) toward SLO‐HRV (GK‐Lika) than in the opposite direction (Appendix S1: Figure S8; Table 2).

**TABLE 2:** BAYESASS estimated migration rates

	Area	Migration to		
		SLO‐HRV(GK‐Lika)	BIH‐HRV(Dal)	SRB
Migration from	SLO‐HRV(GK‐Lika)	0.9643 (0.0118)	0.0229 (0.0105)	0.0128 (0.0088)
	BIH‐HRV(Dal)	0.1958 (0.0320)	0.7664 (0.0355)	0.0378 (0.0175)
	SRB	0.0133 (0.0123)	0.0538 (0.0280)	0.9329 (0.0298)

Note

Standard error of each estimate is noted in parentheses.

### 3.4 Effective population size

We estimated effective population size for each of the clusters identified by STRUCTURE. The results for *N*_E_ were markedly lower for wolves from cluster 1 (Slovenia, Gorski kotar (HRV) and Lika (HRV). The estimated effective population size was higher in the two other clusters (Dalmatia (HRV)‐BIH and Serbia), and we did not detect any statistically significant differences between them. The confidence interval for Serbia was relatively wide, likely due to the much smaller sample size (Table 3).

**TABLE 3:** Results of the effective population size for clusters identified by STRUCTURE

Area	Sample size	*N*_e_ (CI)
SLO‐HRV(Lika‐GK)	112	43.3 (37.9–49.7)
BIH‐HRV(Dal)	106	65.6 (57–76.3)
SRB	37	75.6 (53–123.4)

## 4 DISCUSSION

We observed that wolves in the Dinaric‐Balkan region exhibited northwest–southeast structure, consistent with previous findings for wolves and other taxa in this region (Djan et al., 2014; Glasnović et al., 2018; Šnjegota et al., 2018; Sotiropoulos et al., 2007; Ursenbacher et al., 2008; Veličković et al., 2015). We detected the presence of three genetic clusters in the population, which from northwest to southeast comprise (1) Slovenia and the regions of Gorski kotar and Lika in Croatia, (2) the region of Dalmatia in southern Croatia and BIH, and (3) Serbia. Analyses of the genetic variability showed moderately high genetic variability, albeit slightly lower for wolves from Slovenia and the regions of Gorski kotar and Lika in Croatia. We also detected a substantially lower N_E_ for cluster 1 (Slovenia and the regions of Gorski kotar and Lika in Croatia) relative to the other two clusters.

### 4.1 Population structure, genetic variability, and geneflow

The population structure results were highly consistent among the methods we used, which provides an additional degree of confidence in our findings. We found clear substructure within Croatia, where individuals from Dalmatia showed higher genetic similarity to wolves from BIH, whereas wolves from Lika and Gorski kotar displayed greater similarity to those from Slovenia. This is consistent with results from Fabbri et al. (2014) who detected similar substructure in Croatian wolves, and genetic similarities between Dalmatia and BIH. The authors suggested the effect of climate variations and habitat conditions of Dalmatia, compared to the neighboring higher altitude Dinaric Mountain chain, as potential factors affecting the observed structure. The influence of climate and habitat on wolf population genetic structure has also been suggested in earlier studies of wolves (e.g., Fabbri et al., 2014; Kusak et al., 2000). Fabbri et al. (2014) also detected additional local Lika‐Gorski kotar substructure that seems consistent with Frković and Huber (1993), who noted separate, small populations in these two regions. Our results do not support this additional local substructure, which might be due to the different panels of microsatellites and samples between our study and that of Fabbri et al. (2014), or because Gorski kotar‐Lika substructure has dissolved with increasing wolf numbers and gene flow. The latter scenario also seems supported with the Hardy–Weinberg Dynamic Subsampling analysis (Figure 1b), which indicates a pronounced “isolate breaking” effect of heterozygote excess that can be caused by sampling offspring of immigrants in the recipient cluster, indicating an on‐going mixing of the clusters.

The similarity we observed between wolves from BIH and southern Croatia is consistent with results from Djan et al. (2014). They detected a clear northwest–southeast divergence in mtDNA haplotypes between wolves from Croatia and BIH (their “western” subpopulation) and those from Serbia and North Macedonia (“eastern” subpopulation), which was subsequently confirmed with the same molecular marker, a larger sample, and a broader study area (Šnjegota, 2019, D. Šnjegota, M. Arakelyan, T. Borowik, D. Ćirović, G. Danila, M. Djan, A. Ghazaryan, Z. Gurielidze, T. Hayrapetyan, Z. Hegyeli, A. Karamanlidis, N. Kopaliani, M. Niedziałkowska, K. Plis, D. Politov, A. Vik Stronen, M. Talala, E. Tsingarska, B. Jędrzejewska, unpublished data). The structure we observed between BIH and Serbia is also consistent with earlier microsatellite findings (Šnjegota, 2019), as is the clustering of Croatian and Slovenian wolves, and their differentiation from Balkan region wolves to the south (Greece) and east (Bulgaria) (Montana et al., 2017). Similar results were reported from genome‐wide analyses of SNP markers (Stronen et al., 2013). The observed northwest–southeast gradient thus seems to be supported across multiple studies with three different types of genetic markers.

The statistically significant F_ST_ values detected between wolf clusters, as well as the large number of private alleles found for clusters from BIH/HRV‐Dal and Serbia, further support the observed structuring of wolves in this region. However, examination of more recent profiles from our study area indicated that the high number of private alleles found for the Serbian wolf cluster at locus AHTh260 was due to chance, as subsequent monitoring data show at least two of these six alleles in the Dinaric population (Slovenia, Gorski kotar and Lika) (T. Skrbinšek, unpublished data). We found the lowest genetic variability in cluster 1 (Slovenia, Gorski Kotar and Lika), consistent with the results from Fabbri et al. (2014). This finding, in combination with the low N_E_ detected for cluster 1, might indicate that the wolves in the northwestern Dinaric Mountains passed through a strong bottleneck. Also, being at the edge of the population, this cluster may have received less geneflow from other (sub)population clusters in the past, although the geneflow results indicate it is now receiving considerable geneflow from cluster 2, which is also supported by the heterozygote excess indicated in the cluster 1 area by the HWDS analysis.

As expected, the geneflow analysis indicates more geneflow between the neighboring areas than between more distant areas. The considerably higher migration rate from BIH–HRV (Dalmatia) toward SLO–HRV (GK‐Lika) than in the opposite direction is also interesting, although we find these results difficult to interpret with the current data. The spatial autocorrelation results appear consistent with the STRUCTURE (Figure 3) and sPCA (Figure 4) results in showing genetic structure across our study area, where we would expect positive spatial autocorrelation at shorter distances because of territorial family groups, followed by negative values explained by population genetic structure.

### 4.2 Effective population size

Effective population size is one of the most informative parameters for conservation as it describes both the sensitivity of a population to genetic stochasticity and its evolutionary potential (Waples, 2002). However, this parameter is often difficult to assess, and results must be interpreted carefully. The linkage disequilibrium (LD) method we used assumes discrete generations and no population structure. While we probably came close to meeting the second assumption by estimating Ne separately for each detected population cluster, the assumption of discrete generations is clearly violated. All animals except six individuals from Lika (HRV) were sampled in an 8‐year period, meaning that they should include between 1.7 and 1.9 generations of animals assuming 4.3‐ to 4.7‐year generation time in wolves (Mech et al., 2016). Waples and Do (2010) discuss a reasonable conjecture that if the number of cohorts represented in a sample is approximately equal to the generation length, the LD *N*_E_ estimate should roughly correspond to N_E_ in a generation, which was later supported by Robinson and Moyer (2013). Waples et al. (2014) also showed that mixed‐age adult samples produce *N*_E_ estimates for one generation; however, our samples include a longer time period meaning that more than one cohort of parents may have been included. Because of genetic drift, this causes a temporal genetic structure in the samples and a two‐locus Wahlund effect (mixture LD), which results in a downward bias of the LD estimates of *N*_E_ (Waples et al., 2014). This means that most of our estimates could be biased low, but considering that the timespan for most samples is less than two generations, the actual bias is probably very low.

The estimated effective population sizes for different clusters are low, just around the *N*_E_ > 50 criterion that is considered to allow a population to avoid inbreeding, but still far below the rule‐of‐the‐thumb minimum threshold of *N*_E_ > 500 that has been suggested for maintaining a population's genetic diversity (Allendorf & Luikart, 2009). *N*_E_ is lower for wolves from Slovenia, Gorski kotar, and Lika, as are heterozygosity and allelic diversity. Whereas historic data show that the wolf population in this area was severely reduced in the past, its location at the edge of the larger Dinaric‐Balkan population limits geneflow from other populations, keeping the estimates of genetic diversity parameters and N_E_ lower even if the wolf population is recovering. The long‐term sustainability of this population is vital from a regional and transboundary perspective and requires improved delineation of conservation management units.

Despite increased gene flow between Dinaric and Italian wolves (Ražen et al., 2016), the latter has experienced long‐term isolation and showed comparatively lower genetic diversity than wolves in the Dinaric‐Balkan region (Stronen et al., 2013). Reduction in genetic variability may affect adaptive capacity, particularly when *N*_E_ is low, increasing population vulnerability. Whereas inbreeding has been associated with congenital bone deformities in very small and isolated wolf populations in Scandinavia and on Isle Royale in the United States (Räikkönen et al., 2006, 2009), gene flow appears to have provided a measure of genetic rescue (Scandinavia; Åkesson et al., 2016), at least temporarily in the case of Isle Royale (Hedrick et al., 2014). Given that we did not observe the substructuring between Gorski kotar and Lika wolves detected by Fabbri et al. (2014), our results would appear to support increased gene flow in the northern part of our study area.

### 4.3 Conservation perspective

The Dinaric‐Balkan wolf population is a valuable source of genetic diversity for neighboring populations (Fabbri et al., 2014; Ražen et al., 2016) and shows a considerable level of gene flow between the Caucasus and the Balkans via intermediary populations (Pilot et al., 2014). Moreover, this population is the most transnational in Europe, spanning the largest number of national borders and, consequently, a variety of national monitoring and management approaches (Kaczensky et al., 2013).

The Hardy–Weinberg Dynamic Subsampling analysis results showed considerable wolf geneflow from BIH into Croatia and Slovenia and reduced, but noticeable, geneflow into Serbia, indicating a possible (re)connection of these populations. The results also indicated gene flow among the detected clusters, and BIH may represent a zone of admixture between wolves from clusters 1 and 3. Recent analyses of wolves in Croatia detected wolf‐dog hybrids in Dalmatia, and possible back‐crosses into the wolf population (Kusak et al., 2018) and we cannot exclude the possibility that hybridization and introgression may have affected our findings. However, beyond the initial analysis to detect and remove visible hybrid profiles, future analyses with a larger number of microsatellite or SNP markers will be needed to resolve this issue, which affects several European wolf populations (Salvatori et al., 2020). Nevertheless, the earlier findings from mtDNA (Djan et al., 2014) and genome‐wide SNP profiles (Stronen et al., 2013) found substructure in the Dinaric‐Balkan wolf population unlikely to be explained by wolf‐dog hybridization, and wolf SNP profiles were initially evaluated with dog genotypes to detect possible hybrids (Stronen et al., 2013). Hence, the consistent west‐east divergence observed in analyses of mtDNA, SNPs, and microsatellites support the presence of at minimum two wolf population clusters in the Dinaric‐Balkan region.

Our results may reflect the general trend toward recovery of large carnivores in Europe (Chapron et al., 2014). This increase in numbers may, however, potentially result in conflicts between wolves and humans, leading to over‐hunting. This has been shown to be among the most significant factors affecting wolf populations within Europe (Hindrikson et al., 2017). Human–wolf conflict is particularly important in countries without national wolf legislation, such as BIH, where the observed substructure in the wolf population (Šnjegota, 2019; Šnjegota et al., 2018) might be result of the over‐hunting. Our study showed that wolves from BIH interbreed with those from Dalmatia and may travel northward to Slovenia, where they could contribute to maintaining genetic variability.

Our study adds further support for the west‐east population structure of Dinaric‐Balkan wolves, as previously observed for various taxa across the Balkans (e.g., Djan et al., 2014; Glasnović et al., 2018; Šnjegota et al., 2018; Sotiropoulos et al., 2007; Ursenbacher et al., 2008; Veličković et al., 2015). Numerous factors may contribute to the observed structure, and demographic history, landscape type, prey selection, wolf–dog hybridization, environmental, and ecological factors have all been reported to influence wolf population structure in Europe, North America, and Asia (e.g., Czarnomska et al., 2013; Djan et al., 2014; Jędrzejewski et al., 2012; Koblmüller et al., 2016; Kusak et al., 2000; Muñoz‐Fuentes et al., 2009; Octenjak et al., 2020; Pilot et al., 2006, 2012; Schweizer et al., 2016; Stronen et al., 2015; Werhahn et al., 2018; vonHoldt et al., 2010). Furthermore, recent findings from wolves indicate that functional genetic variation can be linked to important environmental factors such as temperature, precipitation (Schweizer et al., 2016), and elevation (Werhahn et al., 2018). Future genomic analyses could therefore help resolve the observed west‐east population structure and its evolutionary history for wolves and other taxa in the Dinaric‐Balkan region and identify focal sites for habitat conservation in this highly biodiverse area.

## CONFLICT OF INTEREST

The authors declare no conflict of interest.

## AUTHOR CONTRIBUTIONS

**Dragana Šnjegota:** Conceptualization (equal); Data curation (equal); Formal analysis (equal); Writing – original draft (equal); Writing – review & editing (equal). **Astrid Vik Stronen:** Conceptualization (equal); Data curation (equal); Formal analysis (equal); Writing – original draft (equal); Writing – review & editing (equal). **Barbara Boljte:** Data curation (equal); Writing – review & editing (supporting). **Duško Ćirović:** Writing – review & editing (supporting). **Mihajla Djan:** Writing – review & editing (supporting). **Djuro Huber:** Writing – review & editing (supporting). **Maja Jelenčić:** Data curation (supporting); Writing – review & editing (supporting). **Marjeta Konec:** Data curation (equal); Writing – review & editing (supporting). **Josip Kusak:** Writing – review & editing (supporting). **Tomaž Skrbinšek:** Conceptualization (equal); Data curation (equal); Formal analysis (equal); Writing – original draft (equal); Writing – review & editing (equal).

## Supporting information

Supplementary Material

## Data Availability

Data analyzed in this study are available in the Dryad Digital Repository: https://doi.org/10.5061/dryad.ngf1vhhvt.
