# Supplementary material for: Population genetic structure of wolves in the northwestern Dinaric‐Balkan region
Source: Ecol Evol. 2021 Dec 12;11(24):18492–504. doi: 10.1002/ece3.8444 (PMC8717286; doi:10.1002/ece3.8444)
Supplement: Supplementary file 1 — Supplementary Material [file ECE3-11-18492-s001.docx]

**Appendix S1**

**Note S1**

Forty-six dog hair samples from Slovenia (Boljte, 2016: Attachment A) were analysed together with the wolf samples, with the aim of detecting and excluding potential wolf-dog hybrids, using STRUCTURE v2.3.4. Among the 265 putative wolf samples, one individual from Serbia was identified as a dog and nine as wolf-dog hybrids. These ten individuals were removed from further analyses, which were continued with n = 255 samples. The Slovenian, Croatian, and BIH profiles had earlier been examined for wolf-dog hybrids, which have been documented in the study area and represent a concern for the long-term conservation of wolves (Kusak et al., 2018).


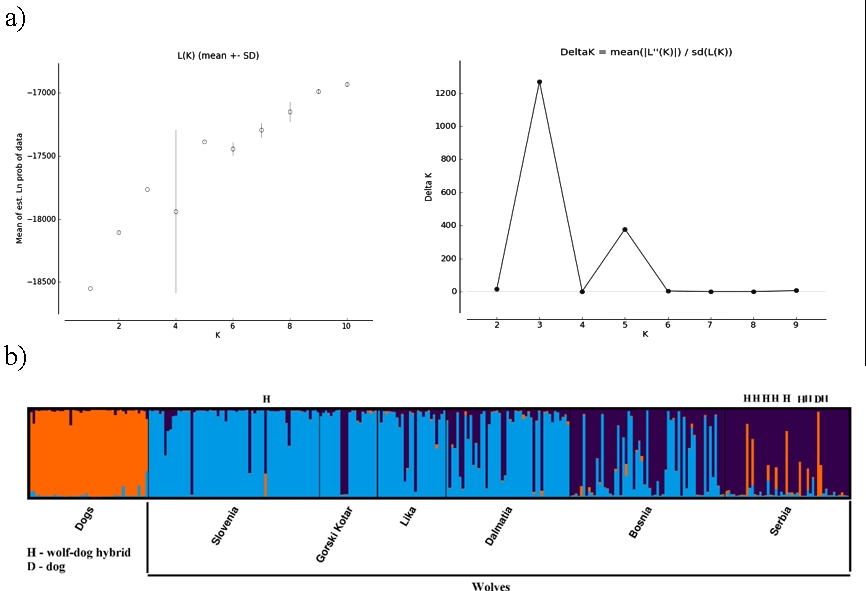


**Figure S1.** a) The most likely number of clusters (K=3) detected for wolves and dogs in STRUCTURE analyses based on log - likelihoods [ln Pr(*X*|*K*)] and the ΔK method b) Bar plot from the initial STRUCTURE analyses showing the clustering of dogs in one genetic cluster and splitting of wolves into two genetic clusters. Each colour corresponds to one cluster; each vertical line represents one individual, showing probability of assignment (range 0-1) per cluster.


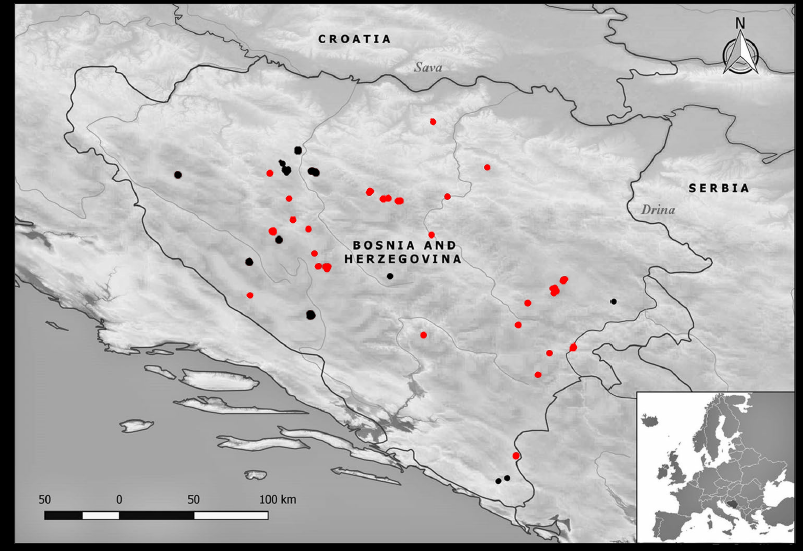


**Figure S2**. Map of samples from Šnjegota et al. (2018), where red dots designate samples used and genotyped *de novo* in this study.


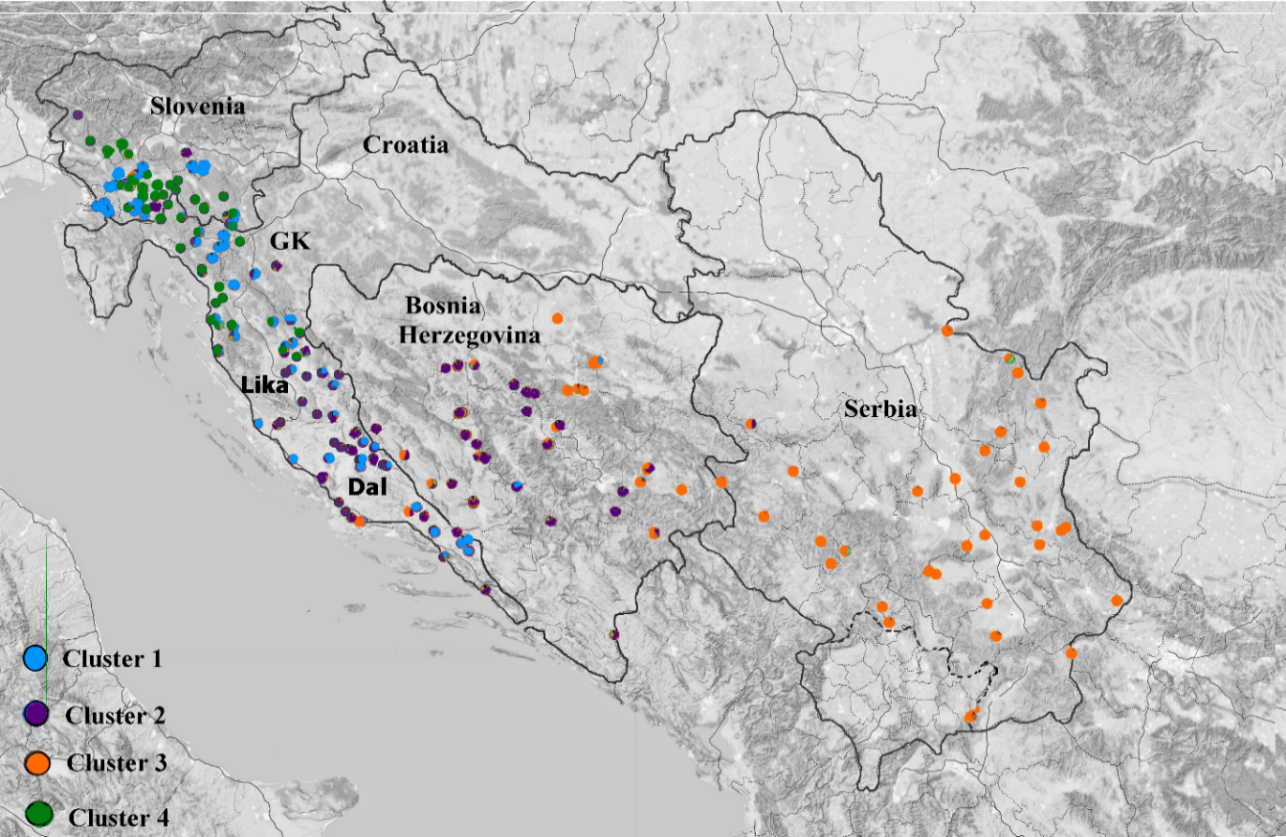


**Figure S3.** The geographical distribution of wolves from STRUCTURE with K=4 population clusters.


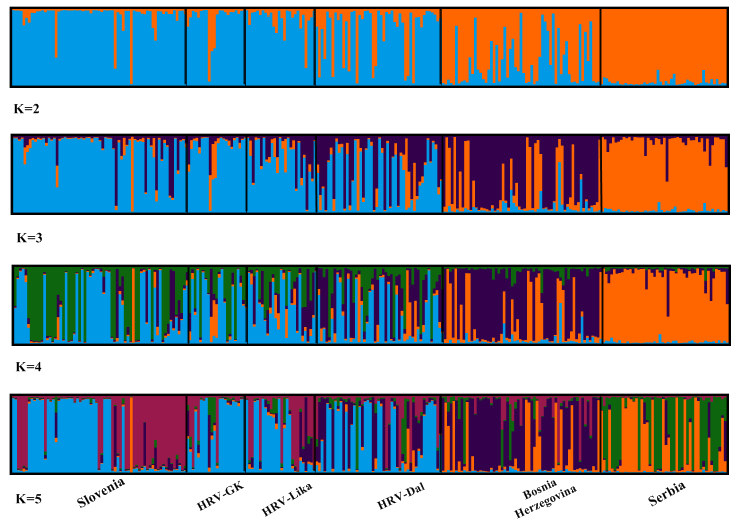


**Figure S4.** Bar plots from the STRUCTURE analyses showing the clustering of wolves in K=2, K=3, K=4, and K=5 genetic clusters. Each colour corresponds to one cluster; each vertical line represents one individual, showing probability of assignment (range 0-1) per cluster.


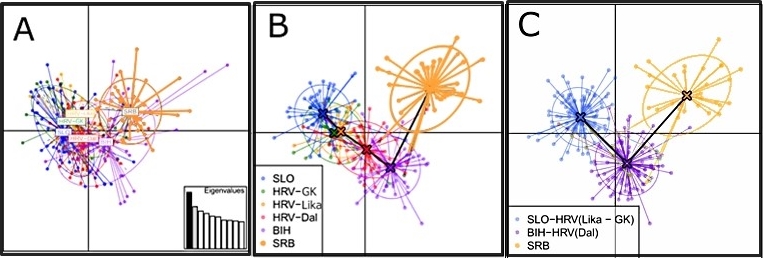


**Figure S5.** PCA and DAPC results: A. PCA axes 1+2; B. DAPC with *a priori* grouping by sampling area; C. DAPC with *a priori* grouping according to STRUCTURE results.

**Figure S6.** Correlogram showing results of the spatial autocorrelation analysis across wolves in the northwestern Dinaric-Balkan region. Lower and upper dotted red lines represent upper and lower limits of a 95% confidence interval for r (spatial autocorrelation coefficient) under the null hypothesis of no autocorrelation between genetic and spatial distances (999 permutations).

**Figure S7.** Number of private alleles detected for the specific loci in the consenus clusters (K = 3).


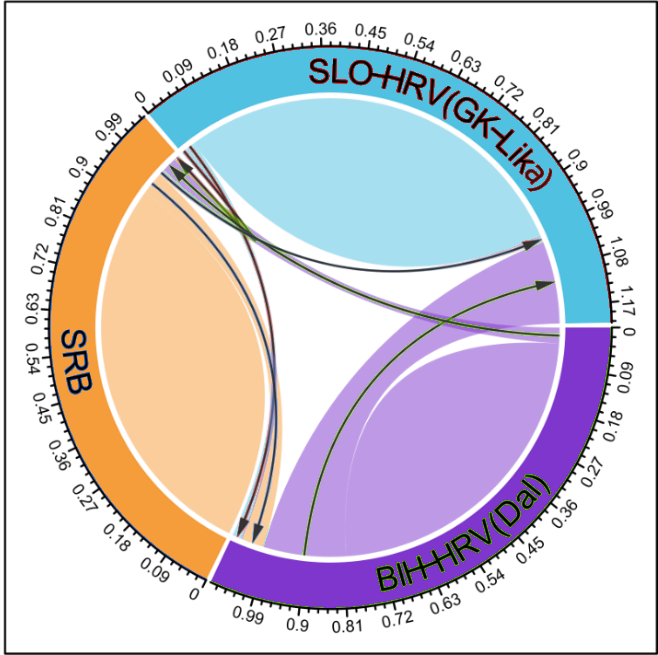


**Figure S8**. D: BAYESASS results. Arrows indicate the direction of migration, the width of the ribbons indicates the migration rates.

**Table S1.** Matrix of the pairwise F_ST_ values between the consensus clusters (K=3), based on the 16 microsatellite loci. Cluster 1 (Slovenia, Gorski Kotar, Lika), cluster 2 (Bosnia & Herzegovina, Dalmatia), cluster 3 (Serbia). All values are statistically significant (p < 0.001).

|  | **Cluster 1** | **Cluster 2** | **Cluster 3** |
| --- | --- | --- | --- |
| **Cluster 1** | 0 |  |  |
| **Cluster 2** | **0.0599** | 0 |  |
| **Cluster 3** | **0.0353** | **0.0445** | 0 |
